# Effect of fenofibrate on residual beta cell function in adults and adolescents with newly diagnosed type 1 diabetes: a randomised clinical trial

**DOI:** 10.1007/s00125-024-06290-6

**Published:** 2024-10-30

**Authors:** Pernille E. Hostrup, Tobias Schmidt, Simon B. Hellsten, Rebekka H. Gerwig, Joachim Størling, Jesper Johannesen, Karolina Sulek, Morten Hostrup, Henrik U. Andersen, Karsten Buschard, Yasmin Hamid, Flemming Pociot

**Affiliations:** 1 Department of Clinical Research, Steno Diabetes Center Copenhagen, Copenhagen University Hospital, Herlev, Denmark; 2 Department of Paediatrics, Copenhagen University Hospital, Herlev and Gentofte, Denmark; 3 The August Krogh Section, Department of Nutrition, Exercise and Sports, University of Copenhagen, Copenhagen, Denmark; 4 Department of Patient Care, Steno Diabetes Center Copenhagen, Copenhagen University Hospital, Herlev, Denmark; 5 The Bartholin Institute, Department of Pathology, Rigshospitalet, Copenhagen, Denmark; 6 Department of Clinical Medicine, Faculty of Health and Medical Sciences, University of Copenhagen, Copenhagen N, Denmark

**Keywords:** Beta cell function, Beta cell stress, Clinical trial, Fenofibrate, Newly diagnosed type 1 diabetes, Peroxisome proliferator-activated receptor alpha (PPARα), Type 1 diabetes

## Abstract

**Aims/hypothesis:**

Fenofibrate, a peroxisome proliferator-activated receptor alpha agonist, shows some promise in alleviating beta cell stress and preserving beta cell function in preclinical studies of type 1 diabetes. The aim of this phase 2, placebo-controlled, double-blinded, randomised clinical trial was to investigate the efficacy and safety of fenofibrate in adults and adolescents with newly diagnosed type 1 diabetes.

**Methods:**

We enrolled 58 individuals (aged 16 to 40 years old) with newly diagnosed type 1 diabetes and randomised them to daily oral treatment with fenofibrate 160 mg or placebo for 52 weeks (in a block design with a block size of 4, assigned in a 1:1 ratio). Our primary outcome was change in beta cell function after 52 weeks of treatment, assessed by AUC for C-peptide levels following a 2 h mixed-meal tolerance test. Secondary outcomes included glycaemic control (assessed by HbA_1c_ and continuous glucose monitoring), daily insulin use, and proinsulin/C-peptide (PI/C) ratio as a marker of beta cell stress. We assessed outcome measures before and after 4, 12, 26 and 52 weeks of treatment. Blinding was maintained for participants, their healthcare providers and all staff involved in handling outcome samples and assessment.

**Results:**

The statistical analyses for the primary outcome included 56 participants (*n*=27 in the fenofibrate group, after two withdrawals, and *n*=29 in the placebo group). We found no significant differences between the groups in either 2 h C-peptide levels (mean difference of 0.08 nmol/l [95% CI −0.05, 0.23]), insulin use or glycaemic control after 52 weeks of treatment. On the contrary, the fenofibrate group showed a higher PI/C ratio at week 52 compared with placebo (mean difference of 0.024 [95% CI 0.000, 0.048], *p*<0.05). Blood lipidome analysis revealed that fenofibrate repressed pathways involved in sphingolipid metabolism and signalling at week 52 compared with placebo. The 52 week intervention evoked few adverse events and no serious adverse events. Follow-up in vitro experiments in human pancreatic islets demonstrated a stress-inducing effect of fenofibrate.

**Conclusions/interpretation:**

Contrary to the beneficial effects of fenofibrate found in preclinical studies, this longitudinal, randomised, placebo-controlled trial does not support the use of fenofibrate for preserving beta cell function in individuals with newly diagnosed type 1 diabetes.

**Trial registration:**

EudraCT number: 2019-004434-41

**Funding:**

This study was funded by the Sehested Hansens Foundation.

**Graphical Abstract:**

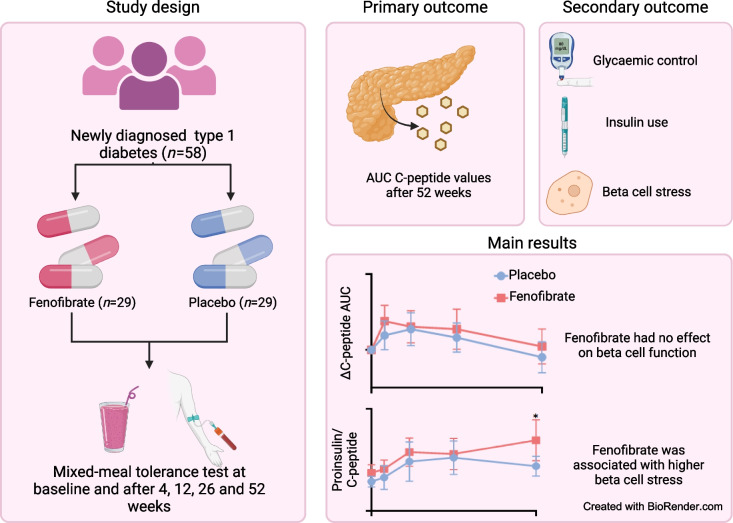

**Supplementary Information:**

The online version contains peer-reviewed but unedited supplementary material available at 10.1007/s00125-024-06290-6.


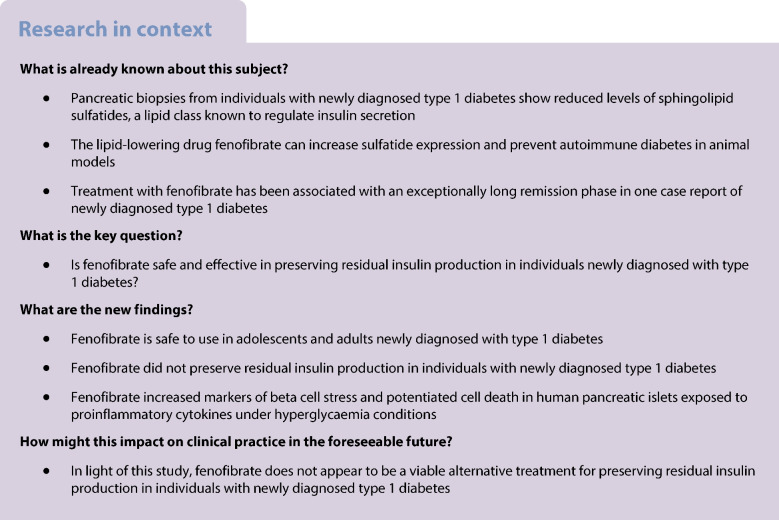


## Introduction

Type 1 diabetes is an autoimmune disease where insulin-producing beta cells of the pancreas are destroyed by infiltrating immune cells, leading to insulin deficiency. More than 8 million individuals worldwide have type 1 diabetes, and the incident rate is increasing [1]. Despite a few clinical trials showing promising effects of immunosuppressive drugs in preserving residual beta cell function, insulin replacement therapy remains the only widely accepted treatment for people with clinical type 1 diabetes (i.e. stage 3 type 1 diabetes). Insulin therapy is highly effective in lowering blood glucose levels, but it is a demanding treatment with considerable adverse effects, such as hypoglycaemia [2]. Therefore, new treatment strategies that can preserve beta cell function and minimise the need for insulin therapy would be of great benefit.

Alterations in sphingolipid metabolism, including repression of the glycosphingolipid sulfatide, have been proposed as a contributing factor to the pathophysiology of type 1 diabetes [3]. This is based on studies showing reduced sulfatide levels in pancreas biopsies of patients with newly onset type 1 diabetes compared with matched controls [4]. Sulfatides play multiple roles in regulating insulin secretion and processing. It interacts with ATP-sensitive potassium channels in the beta cell membrane, causing conformational change to an open state, thereby inhibiting insulin release and relieving beta cell stress [5]. Furthermore, it acts as a chaperone for insulin during its folding [6]. Thus, sulfatide may function as a brake on insulin release during hyperglycaemia to counter beta cell stress [7]. For this reason, sulfatide has been proposed as a putative target to counter beta cell stress and preserve residual insulin production in newly diagnosed type 1 diabetes.

Peroxisome proliferator-activated receptor alpha (PPARα) agonists are known to regulate lipid metabolism, including sulfatides. We recently observed that fenofibrate, a PPARα agonist, completely prevented autoimmune diabetes in NOD mice and reversed diabetes in 50% of NOD mice if administered after disease development. These effects correlated with increased sulfatide expression and reduced T cell migration into pancreatic islets (i.e. insulitis) [4]. Promising as this may seem, the effect of fenofibrate on beta cell function in individuals with newly diagnosed type 1 diabetes remains unknown. A case report from 2020 revealed some potential of fenofibrate by demonstrating maintained euglycaemia, without any insulin therapy, in a patient with newly diagnosed type 1 diabetes treated with a daily oral dose of fenofibrate initiated 7 days after diagnosis [8]. However, longitudinal clinical trials are needed to clarify the effect of fenofibrate.

Here, we investigated the safety and efficacy of fenofibrate in a double-blinded, placebo-controlled, randomised clinical trial in adults and adolescents with newly diagnosed stage 3 type 1 diabetes.

## Methods

### Trial design and oversight

This study was a phase 2, randomised, placebo-controlled, double-blinded trial conducted at the Steno Diabetes Center Copenhagen, Denmark. The trial consisted of a screening period of up to 6 weeks following diagnosis and a treatment period of 52 weeks. Primary and secondary outcomes were assessed at baseline and at 4, 12, 26 and 52 weeks after the initiation of trial treatment.

Before enrolling any participants, the trial protocol was approved by the local ethics committee and the Danish Medicines Agency under the EudraCT number: 2019-004434-41. The trial was conducted in accordance with the principles of the Declaration of Helsinki and Good Clinical Practice. An independent data monitoring committee oversaw the trial.

### Participants, randomisation and treatment

Participants were recruited from Steno Diabetes Center Copenhagen and Bispebjerg Hospital (and other outpatient clinics located in the Capital Region of Denmark) between June 2020 and April 2022. To obtain representation of the broader population of interest both male and female participants were included in the study. Sex was self-reported and cross-referenced with information in the participant’s medical record. Ethnicity, religion and socioeconomic status were not considered as factors of eligibility. However, the recruiting centres are known to serve a diverse patient population in terms of these factors.

All participants were orally informed about the trial and provided with written information before giving written informed consent. Eligible participants were aged 16–40 years, diagnosed with stage 3 type 1 diabetes according to the ADA criteria, and able to undergo randomisation within 6 weeks from the first insulin injection. Other inclusion criteria were the presence of at least one positive diabetes-specific autoantibody, no contraindications to receiving fenofibrate, and a peak C-peptide level >0.2 nmol/l after a 2 h mixed-meal tolerance test (MMTT) (a complete overview of inclusion criteria is provided in electronic supplementary material [ESM] Methods).

In a block design with a block size of 4, 58 participants were randomly assigned in a 1:1 ratio to receive either fenofibrate or placebo. Pre-packed pill containers with identical tablets of fenofibrate or placebo were supplied by the Pharmacy of the Capital Region of Denmark. The randomisation sequence was computer-generated, with fixed numbers of treatments in each block, to balance the treatment allocation over time. Blinding was maintained for participants, their healthcare providers, and all staff involved in handling outcome samples and assessment. The allocation codes were kept in sealed envelopes and remained concealed until the unblinding phase. Fenofibrate was administered as a daily oral dose of 160 mg, based on its pharmacokinetics, pharmacodynamics and safety data, and in alignment with a case report showing preserved beta cell function 2 years after diagnosis in a patient with type 1 diabetes on daily treatment with 160 mg fenofibrate [8].

Participants received individually numbered pill containers at baseline and at 4, 12 and 26 weeks after randomisation. Compliance was assessed by counting the number of returned pills at each trial visit after randomisation.

### Clinical procedures

Trial visits were conducted in the morning following an overnight fast. Long-acting insulin was administered in the evening prior to the visit, and no rapid-acting insulin was administered within 2 hours preceding the start of the visit.

An MMTT was conducted at all visits to assess beta cell function. Participants ingested a standardised liquid meal, and venous blood samples for C-peptide and glucose analyses were collected at 0, 15, 30, 60, 90 and 120 min after the start of ingestion.

Glucose variability was evaluated using a continuous glucose monitoring (CGM) system of the FreeStyle Libre type, which participants wore from baseline to the 52 week follow-up visit. Compliance with the CGM device was assessed at each visit. If the sensor had been active for less than 70% of the time, the data were considered as missing data*.*

The use of exogenous insulin was self-reported by the participants for 7 days leading up to each visit using the FreeStyle Libre app on their mobile device.

### Clinical outcomes

The primary outcome was the change in endogenous insulin production from baseline to after 52 weeks of treatment, assessed by AUC values for C-peptide levels following an MMTT [9].

Predefined secondary outcomes were changes in peak C-peptide following an MMTT; HbA_1c_; glucose outcomes measured with CGM (percentage of time spent with glucose concentrations <3.0, 3.0–3.8, 3.9–10.0, 10.1–13.9 and >13.9 mmol/l, and number of hypoglycaemic events [glucose level <3.9 mmol/l persisting for more than 15 min] [10]); daily insulin dose; percentage of participants in remission at week 52 (HbA_1c_ <53 mmol/mol or 7.0% and an insulin use ≤ 0.4 U kg^-1^ day^-1^) [11]; and beta cell stress assessed by total proinsulin/C-peptide (PI/C) ratio [12]. Proinsulin levels were assessed using a validated ELISA assay. Due to high cross-reactivity between intermediates and intact proinsulin, total proinsulin levels were used for PI/C ratio [cross-reactivity with intact proinsulin of 74% for split (32–33), 65% for des (31, 32), 78% for split (65–66), and 99% for des (64, 65)-proinsulin].

Autoantibodies against GAD65, IA-2, IAA and ZnT8 were detected using the ‘Antibody detection by agglutination-PCR’ method (see ESM Methods). Safety outcomes were assessed by adverse events during the 52 weeks of intervention. At each visit after randomisation, participants were asked about adverse events in general, and specific adverse events known to be associated with fenofibrate.

### Lipid profiling

For lipidomics, plasma samples were combined with NaCl and chloroform/methanol (2:1), after which lipid-containing chloroform was analysed. Data were processed using the open-source software Skyline (see ESM Methods).

### Human islet experiments

Human pancreatic islets were obtained from a population of seven non-diabetic organ donors (see ESM Human Islets Checklist). From these islets, six subpopulations were exposed to proinflammatory cytokines (50 U/ml recombinant human IL-1β and 1000 U/ml recombinant human IFN-γ) with or without fenofibrate before evaluating cell death and three subpopulations were used for glucose-stimulated insulin secretion (GSIS). One subpopulation was lost during the cell death experiment. Cell death was quantified by measuring cytoplasmic histone-associated DNA fragments (see ESM Methods).

### Sample size

We based our sample size on an ANOVA design to detect a between-group difference in 2 h C-peptide AUC after 52 weeks of treatment with either fenofibrate or placebo, with a 1:1 allocation. Our estimates followed the TrialNet Study Group guidelines for ANOVA models in clinical trials involving newly diagnosed type 1 diabetes [13]. Due to the expected skewness in the distribution of C-peptide AUC values, we performed a logarithmic transformation (log[x+1]) to normalise the data. We assumed a geometric-like mean of 0.281 nmol/l in the placebo group at week 52, with variability similar to that reported by the TrialNet Study Group [13]. For a two-tailed test to detect a 50% between-group difference at a 5% significance level and 85% power, our estimates indicated a required sample size of 58 participants, accounting for a dropout rate of no more than 10%.

### Statistical analyses

Data from all participants who received trial treatment and had at least one analysable value available were included in the statistical analyses for both primary and secondary outcomes.

For the primary outcome, we applied a mixed model for repeated measures fitted on log[x+1]-transformed C-peptide AUC data from each visit, with the back-transformed mean value reported as the geometric-like mean (using exp(y) −1). The response variable was post-baseline log[AUC+1] with sex, time and treatment group as fixed effects, participant as random effect, and age at baseline, time to randomisation (defined as the time from first recognised symptoms of hyperglycaemia to the baseline visit) and C-peptide level at baseline as continuous covariates. For participants with incomplete data, missing data were assumed to be missing at random. Missing data were accounted for by using the mixed model for repeated measures.

A similar mixed model approach was used to assess secondary outcomes. For binary dependent variables, a logistic regression model was employed. The model was adjusted for treatment group, sex, age at baseline, and time to randomisation to assess odds ratios (ORs) and 95% CIs. For human pancreatic islet analysis, data were log[x+1]-transformed, and effects were estimated using a mixed linear model.

All reported *p* values are two-sided with a statistical significance level at 5%. Multiple comparisons were adjusted using the Benjamini–Hochberg approach. Statistical analyses were conducted using SPSS software (IBM SPSS Statistics version 25.0, IBM, Armonk, NY, US).

## Results

### Participants

**Fig. 1:**
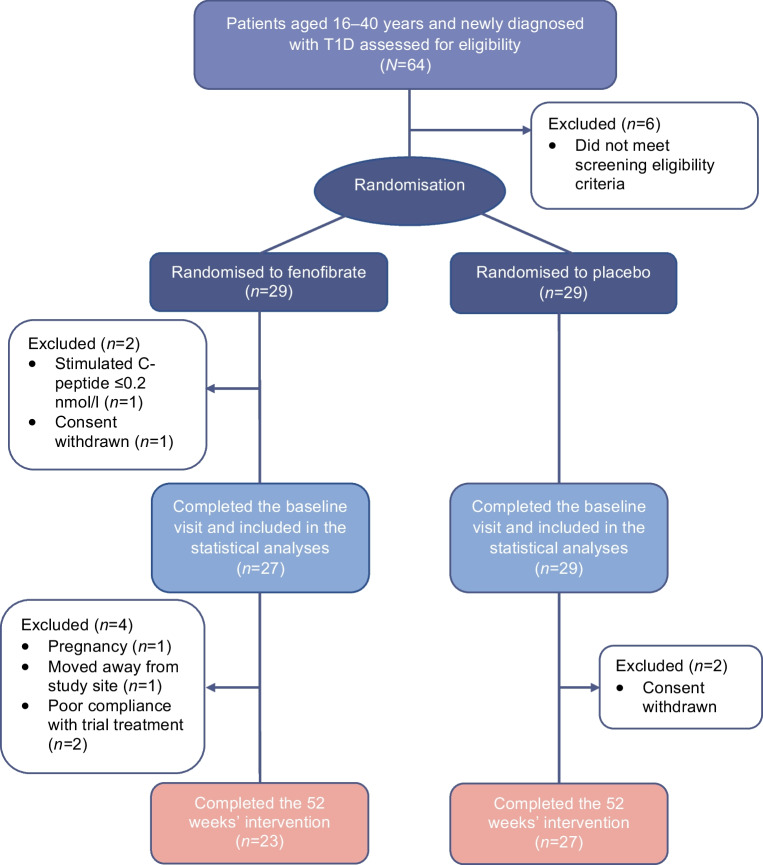
Fifty-eight participants were randomised to 52 weeks of treatment with either fenofibrate 160 mg/day or placebo in a 1:1 ratio. Two participants in the fenofibrate group dropped out after randomisation, but before receiving any trial treatment, and therefore were not included in the statistical analyses. During the 52 weeks of intervention, six participants in the fenofibrate group and two in the placebo group dropped out. T1D, type 1 diabetes

Between July 2020 and April 2022, we assessed 64 individuals newly diagnosed with type 1 diabetes for eligibility. Of these, 58 individuals met the criteria for enrolment and were randomly assigned to receive fenofibrate (*n*=29) or placebo (*n*=29) for 52 weeks. One individual withdrew consent immediately after the baseline visit and did not start the intervention, while another did not achieve a stimulated C-peptide level required for inclusion in the trial (Fig. 1). Consequently, the statistical analyses for the primary outcome included 56 participants (*n*=27 in the fenofibrate group and *n*=29 in the placebo group).

Demographic and clinical baseline characteristics are presented in Table 1. The 2 h C-peptide AUC level was significantly higher in the placebo group at baseline. There was an unequal distribution of participants with diabetic ketoacidosis (DKA) at the time of diagnosis between the groups, with 37% (10 out of 27) in the fenofibrate group compared with 3.4% (1 out of 29) for placebo.

**Table 1:** Baseline characteristics

Characteristic	Fenofibrate *N*=27	Placebo *N*=29
Age, years		
Mean	25.7±5.3	27.2±6.1
Median	26	27
Male sex, *n* (%)	18 (67)	18 (62)
BMI, kg/m^2^	21.5±2.5	22.4±2.4
Disease duration^a^, days	22.0±10.6	24.5±11.3
HbA_1c_, mmol/mol	94±17	91±21
HbA_1c_, %	10.8±1.6	10.5±1.9
Daily insulin dose, U kg^−1^ day^−1^	0.31±0.16	0.28±0.17
2 h C-peptide AUC, nmol/l	0.50±0.20	0.65±0.31
DKA at diagnosis^b^, *n* (%)	10 (37)	1 (3.4)
No. of type 1 diabetes-specific autoantibodies detected at screening^c^, *n* (%)		
1	5 (18.5)	3 (10.3)
2	8 (29.6)	8 (27.6)
3	8 (29.6)	12 (41.4)
4	6 (22.2)	6 (20.7)

Data are presented as mean±SD, median or *n* (%)

^a^Number of days from diagnosis of type 1 diabetes to randomisation

^b^Defined as metabolic acidosis (arterial pH <7.3 and/or standard bicarbonate <18 mmol/l) and blood ketones ≥3 mmol/l

^c^Type 1 diabetes-specific autoantibodies were anti-GAD65, anti-IA-2, anti-insulin and anti-ZnT8

The 52 week follow-up for the last participant was conducted in April 2023. Fifty participants completed the trial intervention, with 23 participants (85%) in the fenofibrate group and 27 participants (93%) in the placebo group, yielding a 14% dropout.

The median drug adherence for participants who completed the intervention was 98.4% (IQR 96.0–99.5) for the fenofibrate group and 98.4% (IQR 96.1–99.4) for placebo, with an individual drug adherence of 88% or greater (ESM Table 1).

### Primary outcome

**Fig. 2:**
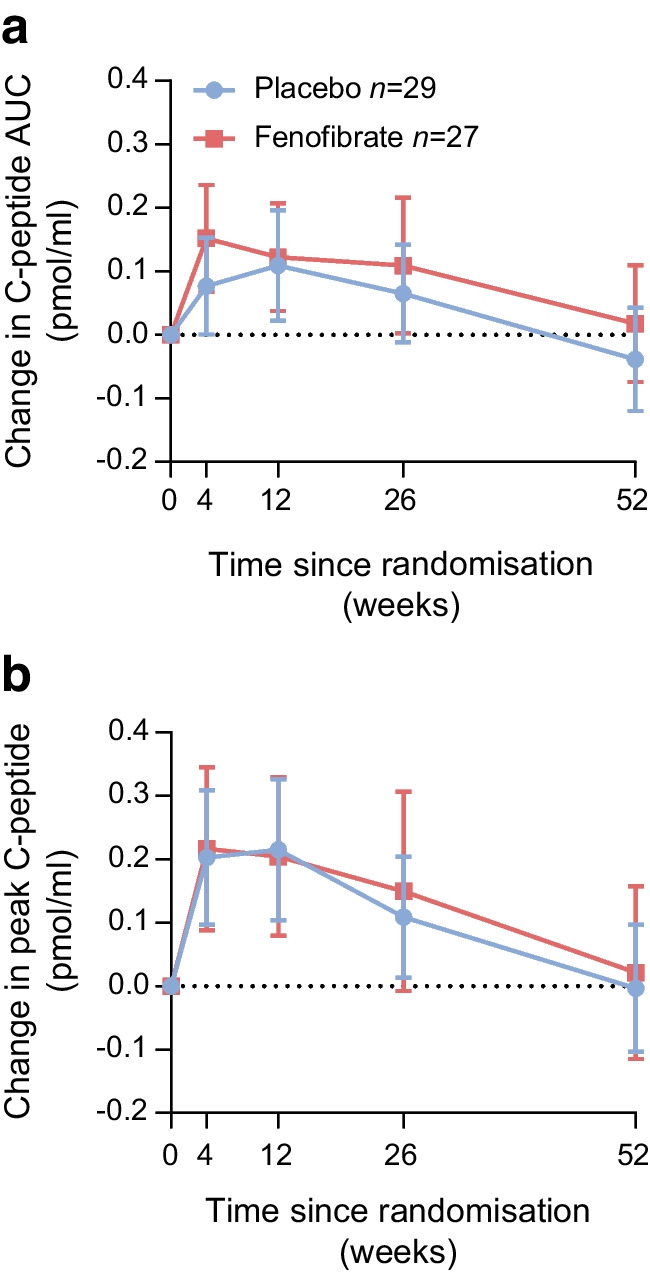
Data are presented as observed mean change from baseline, with 95% CIs, in C-peptide concentrations during a 2 h MMTT over 52 weeks. C-peptide measurements were collected from plasma at the time points 0, 15, 30, 60, 90 and 120 min after ingestion of a standardised meal. (**a**) AUC values calculated from each MMTT using the trapezoid approach. (**b**) Highest C-peptide value obtained during each MMTT. For both (**a**) and (**b**) there were no between-group differences during the 52 weeks of intervention

After 52 weeks of treatment, the mean change from baseline in 2 h C-peptide AUC level was 0.01±0.26 and −0.07±0.23 nmol/l for the fenofibrate group and the placebo group, respectively, with no between-group difference (mean difference of 0.08 nmol/l [95% CI −0.05, 0.23], Fig. 2a, Table 2). When accounting for the stimulated C-peptide level at baseline, the between-group difference was further reduced to 0.005 nmol/l (95% CI −0.10, 0.13).

**Table 2:** Primary and secondary outcomes

Outcome variable	Fenofibrate * N*=23^a^	Placebo *N*=27^a^	Adjusted between-group mean difference (95% CI)	*p* value
Primary outcome at week 52				
Change in C-peptide-AUC geometric-like mean, nmol/l	0.00±0.23	−0.06±0.24	0.08 (−0.05, 0.23)	0.22
Secondary outcomes at week 52				
Change in peak C-peptide, nmol/l	0.02±0.31	−0.00±0.25	0.04 (−0.13, 0.20)	0.68
HbA_1c_, mmol/mol	52±6	50±9	1 (−4, 5)	0.72
HbA_1c_,%	6.9±0.6	6.7±0.8		
Daily insulin dose, U kg^−1^ day^−1^	0.32±0.18	0.27±0.16	0.03 (−0.06, 0.12)	0.50
Participants in partial remission, %	43.5	55.6	OR 0.64 (0.20, 2.07)	0.45
Time spent with a glucose concentration of, %				
>13.9 mmol/l	6±8	6±12	0.6 (−5.9, 7.1)	0.86
10.1–13.9 mmol/l	20±10	17±8	1.9 (−3.5, 7.3)	0.48
3.9–10.0 mmol/l	72±17	76±17	−1.6 (−11.8, 8.6)	0.75
3.0–3.8 mmol/l	1±1	1±1	−0.4 (−1.2, 0.3)	0.23
<3.0 mmol/l	0±0	0±0	NA	NA
PI/C ratio	0.069±0.047	0.043±0.024	0.024 (0.000, 0.048)	0.048*

Data are expressed as mean±SD unless otherwise indicated

^a^A total of 50 participants had data available at week 52. Four participants in the fenofibrate group and two participants in the placebo group had missing data

^*^p values <0.05 were considered statistically significant

NA, not applicable

Nineteen participants (11 from the fenofibrate group and 8 from placebo) had an additional follow-up visit one year after termination of the drug intervention. No significant between-group differences were observed (a mean difference at week 104 of 0.30 nmol/l, [95% CI −0.17, 1.05 nmol/l], ESM Fig. 1). Subgroup analyses based on sex and DKA revealed no significant differences between the fenofibrate group and placebo (ESM Table 2 and ESM Fig. 2).

### Secondary outcomes

#### Peak C-peptide during an MMTT

In alignment with the primary outcome, change in peak C-peptide following an MMTT increased in both groups from baseline until week 12 (Fig. 2b) after which it declined and approximated baseline at week 52 and with no between-group differences (mean difference at week 52 of 0.04 nmol/l [95% CI −0.13, 0.20 nmol/l]).

#### Glycaemic control and insulin dose

**Fig. 3:**
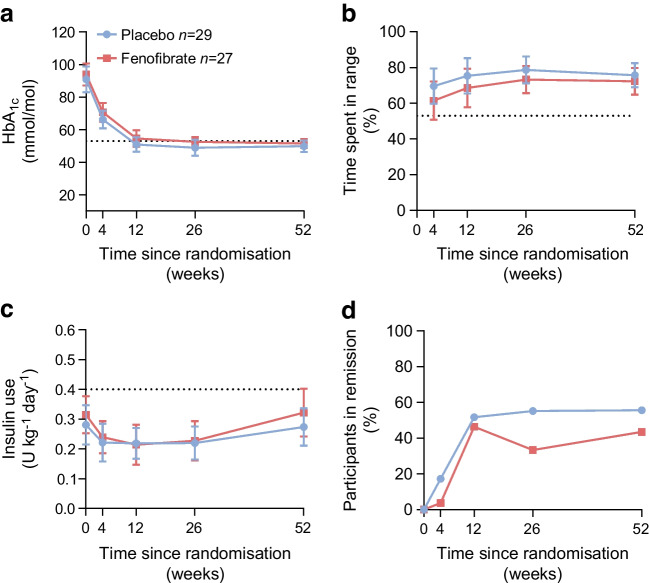
(**a**) HbA_1c_ values for 52 weeks. The dashed line marks the target HbA_1c_ at 53 mmol/mol (7%) for this study population. (**b**) Percentage time spent with a normal blood glucose level (3.9–10.0 mmol/ml), obtained from a CGM device worn continuously for 52 weeks. (**c**) Daily insulin use calculated from self-reported insulin dosage and the participant’s weight on the specific trial visit day during 52 weeks. The dashed line marks an insulin use of 0.4 U kg^−1^ day^−1^. (**a**–**c**) Observed mean values and 95% CIs. (**d**) Percentage of participants in remission at each visit defined as having an HbA_1c_ <53 mmol/mol (7%) and an insulin use ≤0.4 U kg^−1^ day^−1^. (**a**–**d**) No between-group effects during the 52 weeks of intervention

Glycaemic control did not differ between the treatment groups during the intervention (Fig. 3a). HbA_1C_ at baseline was 94±17 mmol/mol (10.8±1.6%) in the fenofibrate group and 91±21 mmol/mol (10.5±1.9%) in the placebo group, and declined for both groups during the intervention, reaching 52±6 mmol/mol (6.9±0.6%) in the fenofibrate group and 50±9 mmol/mol (6.7±0.8%) in the placebo group at week 52, with no between-group differences.

Similarly, the percentage of time spent with a near-normal blood glucose level, assessed by CGM, stabilised around 72±17% for fenofibrate and 76±17% for placebo at week 52, with no between-group difference (Fig. 3b), and the mean insulin dose was comparable between the groups during the 52 weeks’ intervention (mean difference at week 52 of 0.03 U kg^−1^ day ^−1^ [95% CI −0.06, 0.12], Fig. 3c)

#### Remission phase

To address partial remission we adopted the remission criteria outlined by the After Diagnosis Diabetes Research Support System-2 (ADDRESS-2) and used the mid-point values of HbA_1c_ and insulin dose from previous studies addressing remission; i.e. an HbA_1c_ <53 mmol/mol (7.0%) and an insulin use ≤ 0.4 U kg^-1^ day^-1^ [11].

Ten (43.5%) participants in the fenofibrate group and 15 (55.6%) in the placebo group met the set criteria for being in partial remission at week 52 but with no between-group differences (OR for achieving remission in the fenofibrate group compared with placebo: 0.64 [95% CI 0.20, 2.07], Fig. 3d).

#### Beta cell stress

**Fig. 4:**
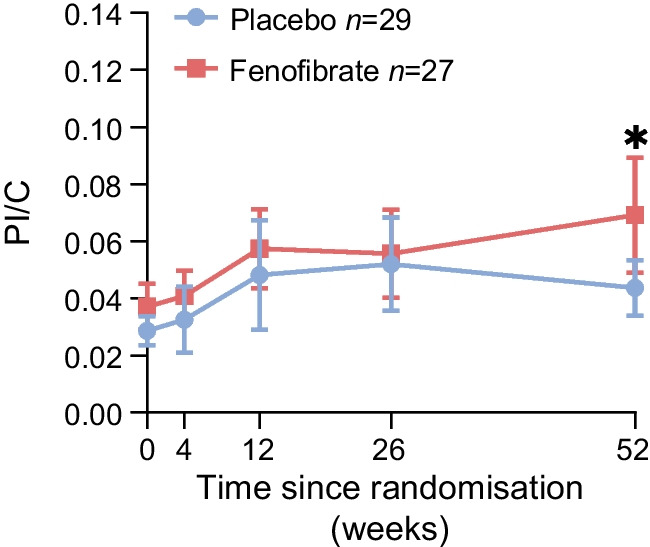
Beta cell stress assessed by the PI/C ratio over 52 weeks. Data represent observed mean values and 95% CIs. Both proinsulin and C-peptide values were obtained from plasma samples collected on trial visit days in the morning after an overnight fast. At week 52, there was a 0.024 (95% CI 0.000, 0.048, *p*<0.05) higher PI/C ratio in the fenofibrate group compared with placebo. *p<0.05

Circulating proinsulin relative to C-peptide is regarded as a valid index for beta cell stress in type 1 diabetes, with elevated PI/C associated with disease progression and treatment responsiveness [14–16]. PI/C increased from 0.027 to 0.052 and 0.038 to 0.056 in the placebo and fenofibrate group, respectively, during the first 26 weeks (Fig. 4), after which it declined to 0.043 by week 52 in the placebo group, but continued to increase in the fenofibrate group, reaching 0.069 by the end of the intervention (between-group difference at week 52 [95% CI 0.000, 0.048], *p*<0.05).

#### Lipid profiling

**Fig. 5:**
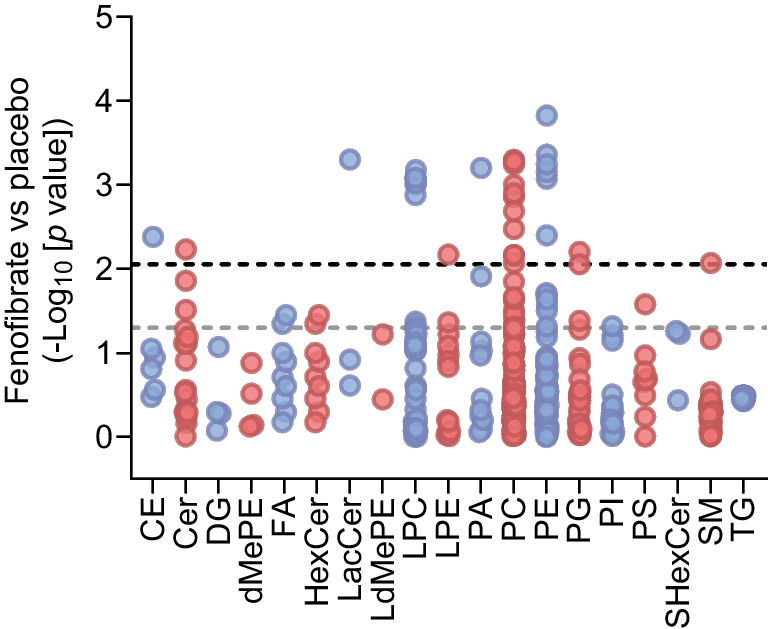
Lipid classes that differed between the fenofibrate group and placebo after 52 weeks of intervention (*n*=27 in the placebo group and *n*=23 in the fenofibrate group). Each dot represents the unadjusted *p* value for a unique lipid species on a −log_10_ scale. The black dashed line marks the FDR-adjusted significance level and the grey dashed line marks a significance level of −log_10_ (0.05). In total 511 unique lipid species were detected, of which 31 reached a *p* value above the FDR-adjusted significance level. Lipid species from the following lipid classes were identified: cholesteryl esters (CE), ceramides (Cer), diglycerides (DG), dimethyl-phosphatidylethanolamines (dMePE), fatty acids (FA), hexosylceramides (HexCer), lactosylceramides (LacCer), lysodimethyl-phosphatidylethanolamines (LdMePE), lysophosphatidylcholines (LPC), lysophosphatidylethanolamines (LPE), phosphatidic acids (PA), phosphatidylcholines (PC), phosphatidylethanolamines (PE), phosphatidylglycerols (PG), phosphatidylinositols (PI), phosphatidylserines (PS), sulfatides (SHexCer), sphingomyelins (SM) and triacylglycerols (TG)

In plasma sampled after an overnight fast at baseline and after 52 weeks, we identified 511 unique lipid species representing 19 different classes. When applying a positive false discovery rate (FDR) of 5% as described by Storey and Tibshirani [17], we observed that 31 lipids were regulated differently between the fenofibrate group and placebo after 52 weeks (Fig. 5). Based on these differently regulated lipids, we next performed an enrichment analysis (https://hyperlipea.org/) of Kyoto Encyclopedia of Genes and Genomes (KEGG) pathway lipids and found that fenofibrate depleted pathways related to sphingolipid metabolism (23.5%), glycerophospholipid metabolism (23.5%), and sphingolipid signalling pathway (17.6%) (Table 3 and ESM Table 3).

**Table 3:** Enrichment analysis of lipid pathways

Pathway name^a^	Converted lipids (%)	*p* values
Sphingolipid metabolism	23.5	0.04007
Glycerophospholipid metabolism	23.5	0.04529
Sphingolipid signalling pathway	17.6	0.04007
Necroptosis	11.8	0.04529
Choline metabolism in cancer	11.8	0.04822

^a^Pathways were identified by an enrichment analysis from lipids significantly different between the intervention groups

*p* values are Benjamini–Hochberg corrected

#### Safety outcomes

Fenofibrate 160 mg was well tolerated in the study cohort, with no serious adverse events reported and no adverse events leading to discontinuation from the trial. In the fenofibrate group, 28 adverse events were recorded, compared with 24 in the placebo group (Table 4). In the fenofibrate group, 25% of reported events were attributed to upper airway infection, 18% to traumas and 29% to transient abnormalities in routine blood analysis. All reported adverse events were mild or moderate (ESM Table 4).

**Table 4:** Safety outcomes

Adverse events during the intervention	Fenofibrate *N*=27	Placebo *N*=29
Adverse events, *n* (% of total events)	28 (54)	24 (46)
Participants with ≥1 event, *n* (% of participants)	18 (67)	13 (45)
Serious adverse events^a^, *n*	0	0
Specific non-serious adverse events, *n* (% of group events)		
Severe hypoglycaemia^b^	1 (3.5)	0
Mild upper airway infection	7 (25)	5 (21)
Headache and other neurological symptoms	4 (14)	2 (8)
Minor traumas	5 (18)	5 (21)
Gastrointestinal symptoms	1 (3.5)	1 (4)
Alopecia	1 (3.5)	0
Muscle pain	1 (3.5)	3 (13)
Abnormal blood analysis^c^	8 (29)	8 (33)

^a^Any untoward medical occurrence or effect that resulted in death, was life-threatening, required hospitalisation or prolongation of existing hospitalisation, resulted in persistent or significant disability or incapacity, or was a congenital anomaly or birth defect

^b^Any documented symptomatic hypoglycaemia or probable symptomatic hypoglycaemia that required assistance of another person

^c^Blood count, platelet count, sodium, potassium, creatinine, creatine kinase, myoglobin, alanine aminotransferase, alkaline phosphatase, international normalised ratio (INR), albumin, amylase, bilirubin and C-reactive protein

### Experiments in human pancreatic islets

**Fig. 6:**
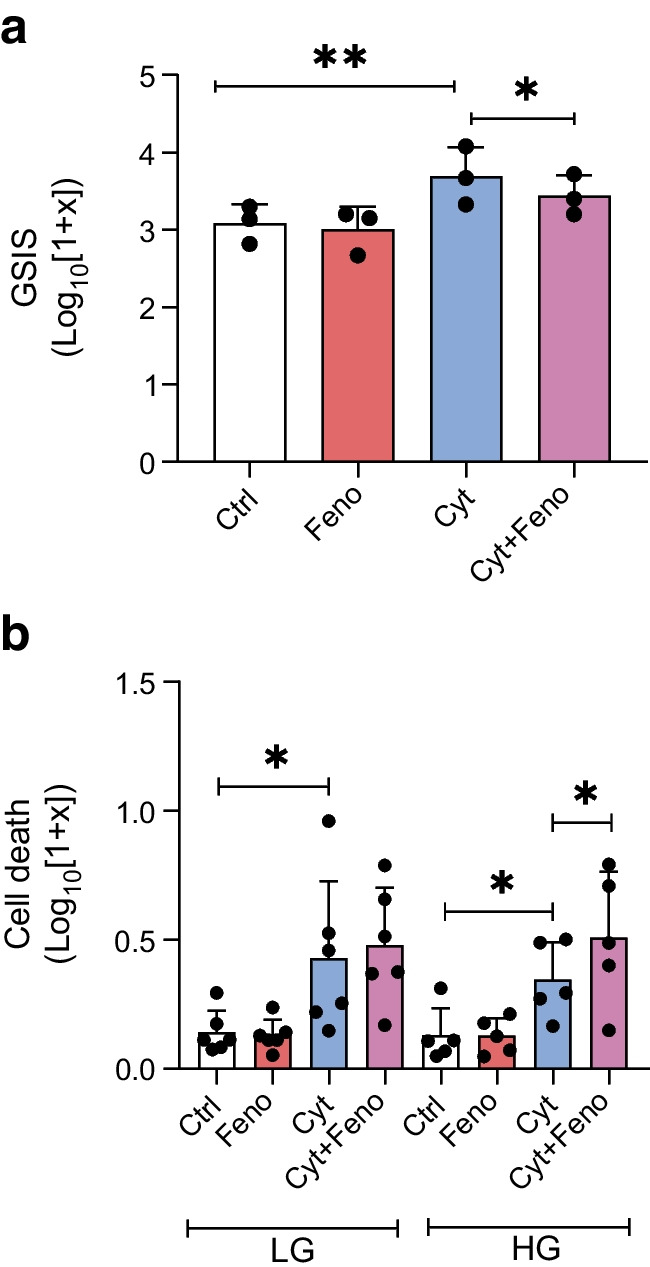
Human pancreatic islets isolated from seven healthy donors, incubated with vehicle (Ctrl), fenofibrate (Feno), proinflammatory cytokines (Cyt) or fenofibrate and proinflammatory cytokines (Cyt+Feno). Data are presented on a Log_10_ (1+x) scale with individual points, representing subpopulations of islets as described in the Methods, together with means and SD. (**a**) GSIS at 20 mmol/l glucose was induced by proinflammatory cytokines (blue) compared with control (white). This effect was diminished in islets co-incubated with fenofibrate and proinflammatory cytokines (purple). (**b**) Cell death was induced by proinflammatory cytokines (blue) at low glucose (LG: 6.1 mmol/l) and high glucose (HG: 16.7 mmol/l), with no effect of fenofibrate alone (red). However, at HG, fenofibrate potentiated the toxic effect of proinflammatory cytokines (purple). *p<0.05, **p<0.01

The protective effects of fenofibrate in NOD mice have been linked to the inhibition of insulin secretion, thereby alleviating beta cell stress [4]. To test this mechanism in humans, we exposed isolated pancreatic islets from human donors to the diabetes-associated proinflammatory cytokines IL-1β and IFNγ [18], and evaluated the effects of fenofibrate on beta cell function and death. Following a 48 h exposure to proinflammatory cytokines, in the presence or absence of fenofibrate, islets were analysed for GSIS during incubation with low (2 mmol/l) or high (20 mmol/l) glucose for 1 h, and the amount of secreted insulin was measured. Compared with untreated control islets, GSIS was not affected in islets exposed to fenofibrate alone (Fig. 6a). Islets exposed to proinflammatory cytokines secreted more insulin than control islets (*p*<0.001, Fig. 6a) [19], an effect that was significantly reduced by fenofibrate (*p*<0.05). Islet cell death was assessed after exposure to cytokines ± fenofibrate at normal (6.1 mmol/l) or high (16.7 mmol/l) glucose for 48 h. Fenofibrate alone did not influence cell death at either high or low glucose concentrations (Fig. 6b). However, fenofibrate augmented cytokine-induced cell death at high (*p*<0.05) but not low glucose.

## Discussion

This study is the first to evaluate the effect of fenofibrate on beta cell function in individuals with newly diagnosed type 1 diabetes. The primary finding was that fenofibrate (160 mg/day) was well tolerated but did not influence beta cell function in adults and adolescents with newly diagnosed type 1 diabetes, as indicated by unchanged C-peptide secretion, insulin usage and glycaemic control. However, a significant secondary finding was that fenofibrate resulted in a higher PI/C ratio than placebo at week 52. Follow-up in vitro experiments in human pancreas islets showed that fenofibrate augmented beta cell death when exposed to proinflammatory cytokines under hyperglycaemic conditions.

Our finding that fenofibrate had no beneficial effect on beta cell function, but even induced a higher PI/C ratio, in a cohort of individuals with newly diagnosed type 1 diabetes underlines the complexity of translating effects from preclinical studies into humans. Preclinical models have indicated that fenofibrate mitigates beta cell stress at hyperglycaemia via mechanisms that reduce insulin secretion [4]. Although we found an inhibitory action of fenofibrate on insulin secretion in human pancreatic islets under conditions that resemble type 1 diabetes (inflammation and hyperglycaemia), this did not relieve beta cell stress but instead induced cell death. Findings in cell lines are also conflicting as fenofibrate has been shown to stimulate rather than inhibit insulin secretion [20, 21]. Such an effect could initially potentiate beta cell function but induce beta cell stress at repeated exposure, and hence explain why we observed a higher PI/C ratio only at week 52. The greater PI/C ratio, along with the detrimental effect observed in human pancreatic islets, questions the applicability of fenofibrate as a protective agent against beta cell stress in newly diagnosed type 1 diabetes.

Given the low expression of sulfatide in pancreas biopsies from six individuals with newly diagnosed type 1 diabetes [4], sulfatide along with other sphingolipids have been suggested to play a role in type 1 diabetes [3]. Notably, we observed that fenofibrate repressed the systemic distribution of plasma lipids involved in sphingolipid signalling and metabolism in people with newly diagnosed type 1 diabetes. While this finding is in contrast to the induction of sulfatides observed in the pancreas of NOD mice exposed to fenofibrate [22], it nevertheless coincides with the systemic lipid-lowering effects of fenofibrate generally reported in human clinical trials [23, 24].

Given that fenofibrate did not affect beta cell function and imposed a few undesirable effects, it is not an evident candidate for multi-drug interventions, which have otherwise been a promising strategy to achieve synergistic effects on residual beta cell function in stage 3 type 1 diabetes [25].

### Strengths and limitations

We enrolled adolescents and adults aged 16–40 years because adult-onset type 1 diabetes constitutes the most prevalent form of the disease [26] but remains under-represented in clinical trials. Furthermore, the remission phase tends to be longer and residual beta cell function maintained in adult-onset type 1 diabetes compared with onset during childhood [11, 27, 28], which potentially would give a better prospect for beta cell preservation upon treatment. In the present study, 56% of the placebo group was still in partial remission at week 52, exceeding that observed in other clinical trials with similar intervention duration in children and adolescents [29, 30]. The long remission phase observed in our cohort could potentially obscure the detection of small drug effects. While we can only speculate on the long-term effects of fenofibrate beyond the first year, a 1 year disease duration is usually sufficient to observe a decline in beta cell function in stage 3 type 1 diabetes and hence commonly applied in clinical trials [9, 28, 31]. For example, in comparable intervention studies performed in adults with similar mean age as in the present study, the control groups exhibited more than a 30% reduction in C-peptide AUC levels 12 months after randomisation [32, 33]. We applied a short screening period of up to 42 days until randomisation to maximise the potential for beta cell rescue. Other trials have typically allowed up to 100 days from diagnosis to randomisation, consequently ending up with a slightly longer disease duration by the end of the intervention.

We experienced a slightly higher than expected dropout rate of 14%, hence affecting the statistical power. However, considering the small effect size of fenofibrate for the primary and several of the secondary clinical outcomes, it seems unlikely that more participants would have changed the overall conclusion of this study. We were able to demonstrate differences in the lipid profile between the two intervention groups, with an overall lipid reduction in the fenofibrate group, which supports a general sufficient drug adherence in this trial.

This study included both male and female participants, providing a representative sample of the broader type 1 diabetes population. Furthermore, sex-specific analyses were conducted; however, no significant sex-driven differences in the effects of fenofibrate were observed.

### Conclusion

In this placebo-controlled, double-blinded study in adults and adolescents with newly diagnosed type 1 diabetes, our findings did not support the previously reported beneficial effects of fenofibrate demonstrated in NOD mice and a single case report. On the contrary, we observed that fenofibrate was associated with higher beta stress in study participants and increased human islet cell death in vitro. Thus, our findings do not support the use of fenofibrate for preserving beta cell function in newly diagnosed type 1 diabetes.

## Supplementary information

Below is the link to the electronic supplementary material.

ESM (PDF 506 KB)

## Data Availability

Data supporting the results from this study are available from the corresponding author on reasonable request.

## Abbreviations

CGM: Continuous glucose monitoring
DKA: Diabetic ketoacidosis
FDR: False discovery rate
GSIS: Glucose-stimulated insulin secretion
MMTT: Mixed-meal tolerance test
PPARα: Peroxisome proliferator-activated receptor alpha
PI/C: Proinsulin/C-peptide

## References

[1] DiMeglio LA, Evans-Molina C, Oram RA (2018) Type 1 diabetes. Lancet 391(10138):2449–2462. 10.1016/S0140-6736(18)31320-5 29916386 10.1016/S0140-6736(18)31320-5 PMC6661119

[2] Hanberger L, Birkebaek N, Bjarnason R et al (2014) Childhood diabetes in the Nordic countries: a comparison of quality registries. J Diabetes Sci Technol 8(4):738–744. 10.1177/1932296814531479 24876421 10.1177/1932296814531479 PMC4764231

[3] Gurgul-Convey E (2020) Sphingolipids in type 1 diabetes: focus on beta-cells. Cells 9(8):1835. 10.3390/cells9081835 32759843 10.3390/cells9081835 PMC7465050

[4] Holm LJ, Krogvold L, Hasselby JP et al (2018) Abnormal islet sphingolipid metabolism in type 1 diabetes. Diabetologia 61(7):1650–1661. 10.1007/s00125-018-4614-2 29671030 10.1007/s00125-018-4614-2 PMC6445476

[5] Buschard K, Blomqvist M, Mansson JE, Fredman P, Juhl K, Gromada J (2006) C16:0 sulfatide inhibits insulin secretion in rat beta-cells by reducing the sensitivity of KATP channels to ATP inhibition. Diabetes 55(10):2826–2834. 10.2337/db05-1355 17003349 10.2337/db05-1355

[6] Buschard K, Bracey AW, McElroy DL et al (2016) Sulfatide preserves insulin crystals not by being integrated in the lattice but by stabilizing their surface. J Diabetes Res 2016:6179635. 10.1155/2016/6179635 26981544 10.1155/2016/6179635 PMC4769769

[7] Marhfour I, Lopez XM, Lefkaditis D et al (2012) Expression of endoplasmic reticulum stress markers in the islets of patients with type 1 diabetes. Diabetologia 55(9):2417–2420. 10.1007/s00125-012-2604-3 22699564 10.1007/s00125-012-2604-3

[8] Buschard K, Holm LJ, Feldt-Rasmussen U (2020) Insulin independence in newly diagnosed type 1 diabetes patient following fenofibrate treatment. Case Rep Med 2020:6865190. 10.1155/2020/6865190 32508930 10.1155/2020/6865190 PMC7245672

[9] Palmer JP, Fleming GA, Greenbaum CJ et al (2004) C-peptide is the appropriate outcome measure for type 1 diabetes clinical trials to preserve beta-cell function: report of an ADA workshop, 21–22 October 2001. Diabetes 53(1):250–264. 10.2337/diabetes.53.1.250 14693724 10.2337/diabetes.53.1.250

[10] Danne T, Nimri R, Battelino T et al (2017) International consensus on use of continuous glucose monitoring. Diabetes Care 40(12):1631–1640. 10.2337/dc17-1600 29162583 10.2337/dc17-1600 PMC6467165

[11] Humphreys A, Bravis V, Kaur A et al (2019) Individual and diabetes presentation characteristics associated with partial remission status in children and adults evaluated up to 12 months following diagnosis of type 1 diabetes: an ADDRESS-2 (After Diagnosis Diabetes Research Support System-2) study analysis. Diabetes Res Clin Pract 155:107789. 10.1016/j.diabres.2019.107789 31326456 10.1016/j.diabres.2019.107789

[12] Scholin A, Nystrom L, Arnqvist H et al (2011) Proinsulin/C-peptide ratio, glucagon and remission in new-onset type 1 diabetes mellitus in young adults. Diabet Med 28(2):156–161. 10.1111/j.1464-5491.2010.03191.x 21219422 10.1111/j.1464-5491.2010.03191.x

[13] Bundy BN, Krischer JP, Type 1 Diabetes TrialNet Study Group (2016) A model-based approach to sample size estimation in recent onset type 1 diabetes. Diabetes Metab Res Rev 32(8):827–834. 10.1002/dmrr.2800 26991448 10.1002/dmrr.2800 PMC5117187

[14] Sims EK, Geyer SM, Long SA, Herold KC (2023) High proinsulin:C-peptide ratio identifies individuals with stage 2 type 1 diabetes at high risk for progression to clinical diagnosis and responses to teplizumab treatment. Diabetologia 66(12):2283–2291. 10.1007/s00125-023-06003-5 37667106 10.1007/s00125-023-06003-5 PMC10914155

[15] Truyen I, De Pauw P, Jorgensen PN et al (2005) Proinsulin levels and the proinsulin:c-peptide ratio complement autoantibody measurement for predicting type 1 diabetes. Diabetologia 48(11):2322–2329. 10.1007/s00125-005-1959-0 16211374 10.1007/s00125-005-1959-0

[16] Loopstra-Masters RC, Haffner SM, Lorenzo C, Wagenknecht LE, Hanley AJ (2011) Proinsulin-to-C-peptide ratio versus proinsulin-to-insulin ratio in the prediction of incident diabetes: the Insulin Resistance Atherosclerosis Study (IRAS). Diabetologia 54(12):3047–3054. 10.1007/s00125-011-2322-2 21959959 10.1007/s00125-011-2322-2

[17] Storey JD, Tibshirani R (2003) Statistical significance for genomewide studies. Proc Natl Acad Sci U S A 100(16):9440–9445. 10.1073/pnas.1530509100 12883005 10.1073/pnas.1530509100 PMC170937

[18] Berchtold LA, Prause M, Storling J, Mandrup-Poulsen T (2016) Cytokines and pancreatic beta-cell apoptosis. Adv Clin Chem 75:99–158. 10.1016/bs.acc.2016.02.001 27346618 10.1016/bs.acc.2016.02.001

[19] Tran DT, Pottekat A, Lee K et al (2022) Inflammatory cytokines rewire the proinsulin interaction network in human islets. J Clin Endocrinol Metab 107(11):3100–3110. 10.1210/clinem/dgac493 36017587 10.1210/clinem/dgac493 PMC10233482

[20] Shimomura K, Ikeda M, Ariyama Y et al (2006) Effect of peroxisome proliferator-activated receptor alpha ligand fenofibrate on K(v) channels in the insulin-secreting cell line HIT-T15. Gen Physiol Biophys 25(4):455–460 17356236

[21] Shimomura K, Shimizu H, Ikeda M et al (2004) Fenofibrate, troglitazone, and 15-deoxy-Delta 12,14-prostaglandin J2 close KATP channels and induce insulin secretion. J Pharmacol Exp Ther 310(3):1273–1280. 10.1124/jpet.104.067249 15201343 10.1124/jpet.104.067249

[22] Holm LJ, Haupt-Jorgensen M, Giacobini JD, Hasselby JP, Bilgin M, Buschard K (2019) Fenofibrate increases very-long-chain sphingolipids and improves blood glucose homeostasis in NOD mice. Diabetologia 62(12):2262–2272. 10.1007/s00125-019-04973-z 31410530 10.1007/s00125-019-04973-z PMC6861358

[23] Camacho-Munoz D, Kiezel-Tsugunova M, Kiss O et al (2021) Omega-3 carboxylic acids and fenofibrate differentially alter plasma lipid mediators in patients with non-alcoholic fatty liver disease. FASEB J 35(11):e21976. 10.1096/fj.202100380RRR 34618982 10.1096/fj.202100380RRR

[24] Peterson LR, Jiang X, Chen L et al (2020) Alterations in plasma triglycerides and ceramides: links with cardiac function in humans with type 2 diabetes. J Lipid Res 61(7):1065–1074. 10.1194/jlr.RA120000669 32393551 10.1194/jlr.RA120000669 PMC7328042

[25] Krogvold L, Mynarek IM, Ponzi E et al (2023) Pleconaril and ribavirin in new-onset type 1 diabetes: a phase 2 randomized trial. Nat Med 29(11):2902–2908. 10.1038/s41591-023-02576-1 37789144 10.1038/s41591-023-02576-1 PMC10667091

[26] Gregory GA, Robinson TIG, Linklater SE et al (2022) Global incidence, prevalence, and mortality of type 1 diabetes in 2021 with projection to 2040: a modelling study. Lancet Diabetes Endocrinol 10(10):741–760. 10.1016/S2213-8587(22)00218-2 36113507 10.1016/S2213-8587(22)00218-2

[27] Davis AK, DuBose SN, Haller MJ et al (2015) Prevalence of detectable C-peptide according to age at diagnosis and duration of type 1 diabetes. Diabetes Care 38(3):476–481. 10.2337/dc14-1952 25519448 10.2337/dc14-1952

[28] Hao W, Gitelman S, DiMeglio LA, Boulware D, Greenbaum CJ, Type 1 Diabetes TrialNet Study Group (2016) Fall in C-peptide during first 4 years from diagnosis of type 1 diabetes: variable relation to age, HbA1c, and insulin dose. Diabetes Care 39(10):1664–1670. 10.2337/dc16-0360 27422577 10.2337/dc16-0360 PMC5033079

[29] Quattrin T, Haller MJ, Steck AK et al (2020) Golimumab and beta-cell function in youth with new-onset type 1 diabetes. N Engl J Med 383(21):2007–2017. 10.1056/NEJMoa2006136 33207093 10.1056/NEJMoa2006136

[30] Forlenza GP, McVean J, Beck RW et al (2023) Effect of verapamil on pancreatic beta cell function in newly diagnosed pediatric type 1 diabetes: a randomized clinical trial. JAMA 329(12):990–999. 10.1001/jama.2023.2064 36826844 10.1001/jama.2023.2064 PMC9960020

[31] Marcovecchio ML, Hendriks AEJ, Delfin C et al (2024) The INNODIA Type 1 Diabetes Natural History Study: a European cohort of newly diagnosed children, adolescents and adults. Diabetologia. 10.1007/s00125-024-06124-5 38517484 10.1007/s00125-024-06124-5 PMC11058619

[32] von Herrath M, Bain SC, Bode B et al (2021) Anti-interleukin-21 antibody and liraglutide for the preservation of beta-cell function in adults with recent-onset type 1 diabetes: a randomised, double-blind, placebo-controlled, phase 2 trial. Lancet Diabetes Endocrinol 9(4):212–224. 10.1016/S2213-8587(21)00019-X 33662334 10.1016/S2213-8587(21)00019-X

[33] Ovalle F, Grimes T, Xu G et al (2018) Verapamil and beta cell function in adults with recent-onset type 1 diabetes. Nat Med 24(8):1108–1112. 10.1038/s41591-018-0089-4 29988125 10.1038/s41591-018-0089-4 PMC6092963

