# Supplementary material for: Effect of fenofibrate on residual beta cell function in adults and adolescents with newly diagnosed type 1 diabetes: a randomised clinical trial
Source: Diabetologia. 2024 Oct 30;68(1):29–40. doi: 10.1007/s00125-024-06290-6 (PMC11663161; doi:10.1007/s00125-024-06290-6)
Supplement: Supplementary file 1 — ESM (PDF 506 KB) [file 125_2024_6290_MOESM1_ESM.pdf]

ESM Methods

Eligibility criteria for enrollment

Inclusion criteria

1. Diagnosed with T1D and aged 16 to 40 years at inclusion
2. First injection of insulin a maximum of six weeks prior to inclusion
3. Must be willing and capable of taking the study drugs and attending tests as described in the protocol
4. Signed informed consent and expected cooperation of the patient for the treatment and follow-up must be obtained and documented according to the guidelines for Good Clinical Practice (GCP) and Danish regulations

Exclusion criteria

1. Treatment with any oral or injected anti-diabetic medications other than insulin
2. A medical history of liver disease, pancreatitis, kidney disease, myositis, or venous thromboembolic events
3. Participation in interventional or other drug research studies which could affect the objectives of this study
4. Inability or unwillingness to comply with the provisions of this protocol
5. Females who are lactating, pregnant, or planning to become pregnant within 12 months after inclusion. Males or fertile females not willing to use adequate contraception if sexually active
6. Presence of a serious disease or condition which, in the opinion of the Investigator, makes the patient ineligible for the study
7. C-peptide levels < 0.2 nmol/l upon inclusion, measured during a MMTT
8. Absence of islet autoantibodies (GAD65, IA-2, IAA, and ZnT8)

Autoantibody analysis

At baseline serum samples were tested for the presence of autoantibodies against GAD65, IA-2, IAA, and ZnT8 with the " Antibody detection by agglutination-PCR" method (ADAP T1Define 2.0^{TM}-serum, Enable Biosciences, USA) [1]. The assay was conducted according to the manufacturer's instructions, using robot-assisted sample preparation (BRAVO robot, Agilent, USA).

Lipid profiling

Plasma samples (10 µl) were combined with 10 µl of 0.9% w/v NaCl (aq) and 120 µl chloroform/methanol (2:1) mixture with internal standards, previously specified in protocols used at Steno Diabetes Center Copenhagen [2]. The lipid-containing chloroform was subjected to analysis using an Agilent ultra-high performance liquid chromatograph coupled with a quadrupole time-of-flight mass spectrometer (UHPLC-QTOFMS, Agilent). The samples were analysed in a randomised order, with quality control (QC) pooled plasma samples interspersed at regular intervals throughout the run (n=10, for both positive and negative ionisation). The lipidomics data underwent pre-processing with Skyline [3], where peaks were cross-matched with an in-house library, which has been developed and validated previously across 31 European laboratories using the National Institute of Standards and Technology Standard Reference Material of human plasma metabolites [4]. Data before statistical analysis were normalised to internal standards and batch-corrected using K-Nearest Neighbors algorithm in R (https://www.r-project.org/).

Human islet experiments

Isolated human pancreatic islets from 7 non-diabetic organ donors were purchased from Prodo Laboratories Inc., Aliso Viejo, CA, USA. Islets were maintained in an F-10 Nutrient Mix medium with GlutaMAX supplemented with 10% fetal bovine serum (FBS), and 1% penicillin/streptomycin. Twenty-five islets in duplicates were exposed for 24 or 48 hours to proinflammatory cytokines (50 U/ml recombinant human IL 1β (R&D Systems, Minneapolis, MN, USA) and 1000 U/ml recombinant human IFN-γ (PeproTech, Cranbury, NJ, USA)) with or without 10 µmol/l Fenofibrate (Cayman

Chemical, Michigan, USA) or DMSO at 6.11 or 16.7 mmol/l glucose before evaluation of cell death or glucose-stimulated insulin secretion (GSIS).

Cell death was quantified by measurement of cytoplasmic histone-associated DNA fragments using The Cell Death Detection ELISA^{PLUS} kit (Roche, Basel, Schwitcherland). Data was normalised to total DNA in nuclei after ultrasound sonication using the Quantiflour® dsDNA System (Promega, Madison, WI, USA).

For GSIS assay, islets were incubated in sterile filtered Krebs Ringer buffer (pH 7.4) with 1 M HEPES (Gibco by Thermo Fisher Scientific, Massachusetts, USA), 7,5 % NaHCO3(Sigma-Aldrich, Missouri, USA) and 0.2% Low endotoxin Bovine Serum Albumin (Sigma-Aldrich, Missouri, USA), with either 2 or 20 mmol/l D-glucose (Sigma-Aldrich, Missouri, USA) with or without cytokines and fenofibrate and then incubated for 30 minutes. Accumulated insulin in the Krebs Ringer buffer was quantified by ELISA (Mercodia) and normalised to DNA content as above.

References

[1] Cortez FJ, Gebhart D, Robinson PV, et al. (2020) Sensitive detection of multiple islet autoantibodies in type 1 diabetes using small sample volumes by agglutination-PCR. PLoS One 15(11): e0242049. 10.1371/journal.pone.0242049

[2] Tofte N, Suvitaival T, Ahonen L, et al. (2019) Lipidomic analysis reveals sphingomyelin and phosphatidylcholine species associated with renal impairment and all-cause mortality in type 1 diabetes. Sci Rep 9(1): 16398. 10.1038/s41598-019-52916-w

[3] Kirkwood KI, Pratt BS, Shulman N, et al. (2022) Utilizing Skyline to analyze lipidomics data containing liquid chromatography, ion mobility spectrometry and mass spectrometry dimensions. Nat Protoc 17(11): 2415-2430. 10.1038/s41596-022-00714-6

[4] Bowden JA, Heckert A, Ulmer CZ, et al. (2017) Harmonizing lipidomics: NIST interlaboratory comparison exercise for lipidomics using SRM 1950-Metabolites in Frozen Human Plasma. J Lipid Res 58(12): 2275-2288. 10.1194/jar.M079012

ESM Table 1: Drug adherence[a]

Fenofibrate *N*=23	Placebo *N*=27
**Median adherence, % (IQR)**	
98.4 (96.0-99.5)	98.4 (96.1-99.4)
**Drug adherence for each participant[b], %**	
92	100
94	99
100	100
99	100
100	98
96	98
99	99
99	100
100	95
98	100
99	99
95	97
96	97
99	98
98	97
99	94
97	99
97	91
98	100
88	90
99	95
92	99
100	96
-	99
-	98
-	95
-	96

[a]Drug adherence for participants who completed 52 weeks of intervention

[b]% days with drug adherence, based on the number of returned pills

ESM Table 2: Subgroup analysis for sex

Outcome variables[a]	Adjusted between-group mean difference at week 52 (95% CI)	*p* value[b]
Change in C-peptide-AUC[c], nmol/l		0.50
Male	0.12 (-0.05 to 0.31)	
Female	0.05 (-0.14 to 0.29)	
Change in peak C-peptide[c], nmol/l		0.48
Male	0.11 (-0.10 to 0.32)	
Female	-0.42 (-0.31 to 0.22)	
HbA_{1c}, mmol/mol		0.24
Male	3 (-3 to 8)	
Female	-1 (-8 to 6)	
Daily insulin dose, U kg^{-1} day^{-1}		0.36
Male	0.05 (-0.38 to 0.16)	
Female	0.01 (-0.13 to 0.15)	
Participants in partial remission OR[d]		0.26
Male	0.50 (0.12 to 2.19)	
Female	1.13 (0.16 to 8.06)	
Time spent with a glucose concentration of 3.9-10.0 mmol/l, %		0.99
Male	-6.8 (-19.3 to 5.8)	
Female	3.6 (-12.6 to 19.7)	
Proinsulin/C-peptide ratio		0.82
Male	0.013 (-0.015 to 0.041)	
Female	0.035 (-0.003 to 0.074)	

[a]Exploratory subgroup analysis for sex, performed posthoc. Estimates of effect were assessed using a mixed model for repeated measures unless otherwise indicated

[b]*p* values represent the interaction between the intervention group and sex

[c]C-peptide concentration in plasma during a 2-hour MMTT over 52 weeks. C-peptide measurements were collected at 0, 15, 30, 60, 90, and 120 minutes. The AUC was calculated using the trapezoid approach. Peak C-peptide values represent the highest C-peptide concentration obtained during each MMTT

[d]Partial remission was defined as having an HbA_{1c} < 53 mmol/mol (7%) and an insulin use ≤ 0.4 U kg^{-1} day^{-1} over 52 weeks. Estimates of effects were assessed by logistic regression and presented as odds ratio (OR) and 95% confidence intervals

ESM Table 3: Alterations in lipidomic profile

Lipid species	Between-group mean difference (95% CI)	*p* value (unadjusted)	*p* value (adjusted)[a]
PE(43:6)	-0.489 (-0.465-0.513)	0.000	0.025
PE(40:6)_A	-0.008 (-0.007-0.008)	0.000	0.037
LacCer(d32:1)	-0.458 (-0.431-0.485)	0.001	0.028
PC(38:6)A	-0.288 (-0.272-0.303)	0.001	0.021
PC(40:8)	-0.043(-0.041-0.045)	0.001	0.018
PC(40:5)B	-0.126(-0.119-0.13)	0.001	0.016
PE(P-42:6)	-0.002(-0.001-0.002)	0.001	0.014
PA(44:4)	-0.333(-0.315-0.351)	0.001	0.013
LPC(22:6)	-0.012(-0.011-0.012)	0.001	0.013
PE(42:6)	-0.038(-0.037-0.039)	0.001	0.012
LPC(22:6/0:0)	-0.010(-0.010-0.011)	0.001	0.013
LPC(0:0/22:6)	-0.010(-0.010-0.011)	0.001	0.012
PE(40:6)	-0.016(-0.015-0.017)	0.001	0.011
LPC(18:0/0:0)	-0.128(-0.120-0.136)	0.001	0.011
LPC(0:0/18:0)	-0.127(-0.119-0.134)	0.001	0.011
PC(38:6)_B	-0.401(-0.377-0.426)	0.001	0.011
PC(40:4)	-0.022(-0.021-0.024)	0.001	0.013
LPC(18:0)	-0.120(-0.112-0.128)	0.001	0.012
PC(38:5)_A	-0.054(-0.051-0.057)	0.001	0.012
PC(40:6)	-0.032(-0.030-0.033)	0.002	0.017
PC(38:3)	-0.132(-0.123-0.140)	0.003	0.027
PE(39:6)	-0.010(-0.009-0.010)	0.004	0.031
CE(18:2)	-0.015(-0.014-0.016)	0.004	0.031
Cer(d40:2)	-0.001(-0.001-0.001)	0.006	0.042
PG(38:2)	-0.011(-0.010-0.011)	0.006	0.042
LPE(P-18:0)	-0.001(-0.001-0.001)	0.007	0.044
PC(P-40:6)/PC(O-40:7)_A	-0.002(-0.002-0.003)	0.007	0.042
PC(40:5)_A	-0.023(-0.021-0.024)	0.007	0.041
PC(P-40:5)/PC(O-40:6)	-0.009(-0.008-0.010)	0.009	0.050
SM(d33:0)	0.000(0.000-0.000)	0.009	0.048
PG(38:3)	-0.014(-0.013-0.015)	0.009	0.048

[a]*p*-values adjusted for positive false discovery using the Storey & Tibshirani approach

ESM Table 4: Safety outcomes

Adverse events during the intervention	Fenofibrate N=27	Placebo N= 29
Adverse events, no. (% of 52 total events)	28 (54)	24 (46)
Participants with one or more events, no. (% of participants)	18 (67)	13 (45)
Serious adverse events, no.	0	0
Diabetic ketoacidosis, no.	0	0
Specific nonserious adverse events, no. (% of group events)		
Severe hypoglycemia	1 (3.5)[a,d]	-
Mild upper airway infection	7 (25)[a,d]	5 (21)[a,d]
Headache and other neurological symptoms	4 (14)[a,d]	2 (8)[a,d]
Minor traumas	5 (18)[a,b,d]	5 (21)[a,d]
Gastrointestinal symptoms	1 (3.5)[a,d]	1 (4)[a,d]
Alopecia	1 (3.5)[a,d]	-
Muscle pain	1 (3.5)[a,d]	3 (13)[a,d]
Abnormal blood analysis	8 (29)	8 (33)
Elevated ALAT	1[a,e]	-
Elevated bilirubin	-	2[a,e]
Elevated creatine kinase	4[a,e]	4[a,e]
Elevated/low blood count	3[a,e]	2[a,e]

Seriousness of the reported adverse events:

[a]Mild: Awareness of signs or symptoms, but no disruption of usual activity

[b]Moderate: Events sufficient to affect the usual activity

[c]Severe: Inability to work or perform usual activities

Action taken to adverse event:

[d]No action taken

[e]Interruption (e.g. control blood sample)

[f]Withdrawn (no cases)

ESM Fig. 1

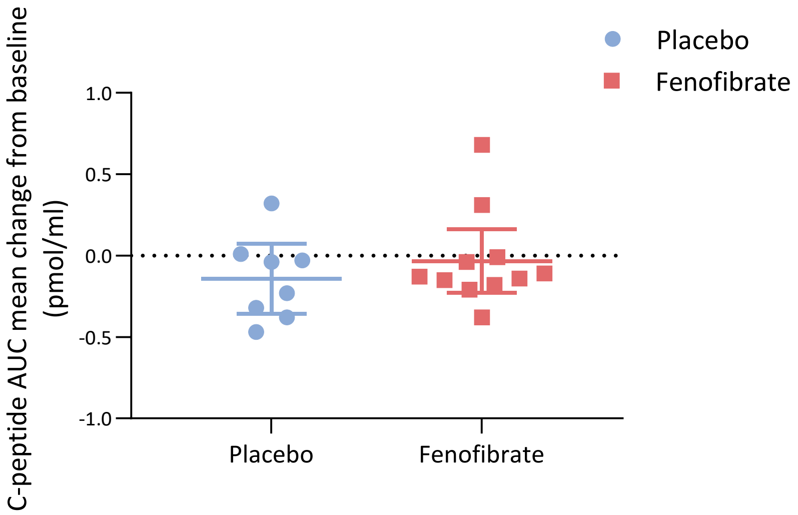

ESM Fig. 1 Data is presented as observed mean change from baseline with 95% CIs in C-peptide concentrations in plasma during a 2-hour MMTT at week 104. C-peptide measurements were collected at the time points; 0, 15, 30, 60, 90, and 120 minutes. The trapezoid approach was used to calculate the AUC values from each MMTT. Estimates of effects were assessed by using a mixed model for repeated measures. The between-group difference at week 104 was 0.30 nmol/l, (95% CI -0.17 to 1.05 nmol/l), *p*=0.20

ESM Fig. 2

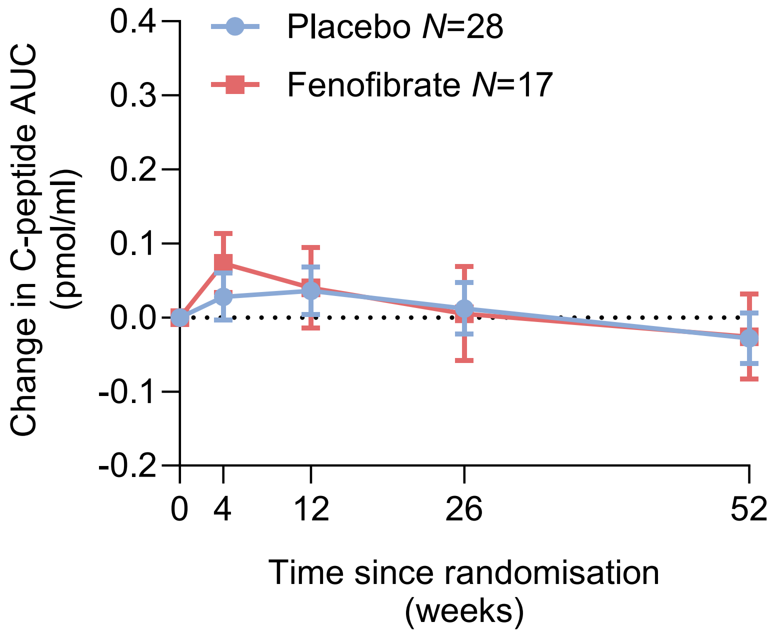

ESM Fig. 2 Comparisons of means for the primary outcome without cases with diabetic ketoacidosis at the time of diagnosis. Data is presented as observed mean change from baseline, with 95% CIs, in C-peptide AUC following an MMTT. There were no mean differences in the primary outcome between the fenofibrate group and placebo when removing all cases with diabetic ketoacidosis from the original analysis

Human Islets Checklist

Checklist for reporting human islet preparations used in research

Adapted from Hart NJ, Powers AC (2018) Progress, challenges, and suggestions for using human islets to understand islet biology and human diabetes. Diabetologia https://doi.org/10.1007/s00125-018-4772-2

**Islet preparation**	**1**	**2**	**3**	**4**	**5**	**6**	**7**
**MANDATORY INFORMATION**							
Unique identifier	HP-21055-01	HP-21132-01	HP-21161-01	HP-21167-01	HP-21197-01	HP-21260-01	HP-21266-01
Donor age (years)	36	31	43	46	53	44	62
Donor sex (M/F)	Female	Male	Male	Male	Male	Female	Female
Donor BMI (kg/m^2)	31.6	26.6	25.5	28.1	32.4	25	28.5
Donor HbA_{1c} or other measure of blood glucose control	5.6	5.2	5.4	5.0	5.5	5.5	5.7
Origin/source of islets[b]	Prodo Labs	Prodo Labs	Prodo Labs	Prodo Labs	Prodo Labs	Prodo Labs	Prodo Labs
Islet isolation centre							
Donor history of diabetes? Please select yes/no from drop down list	No	No	No	No	No	No	No
**If Yes, complete the next two lines if this information is available**							
Diabetes duration (years)							
Glucose-lowering therapy at time of death[c]							
**RECOMMENDED INFORMATION**							
Donor cause of death	Stroke	Anoxic event	Head trauma	Stroke	Anoxic event	Anoxic event	Head trauma
Warm ischaemia time (h)							
Cold ischaemia time (h)							
Estimated purity (%)	90	90	90	85	90	85	85
Estimated viability (%)	95	95	95	95	95	95	95

[b]For example, IIDP, ECIT, Alberta IsletCore

[c]Please specify the therapy/therapies

[d]Time of islet culture at the isolation centre, during shipment and at the receiving laboratory

[e]Please specify the test and the results
